# Risk factor analysis of nodal and distant metastasis for G1 and G2 gastric neuroendocrine tumors

**DOI:** 10.1097/MD.0000000000051423

**Published:** 2026-10-30

**Authors:** Yuhou Shen, Yue Zhang, Lu Liu, Aimin Yue

**Affiliations:** a Department of Surgical Oncology, Xinxiang Central Hospital, The Fourth Clinical College of Xinxiang Medical University, Xinxiang Key Laboratory of Tumor Biotherapy, Xinxiang, China; b Department of Colorectal Surgery, National Cancer Center/National Clinical Research Center for Cancer/Cancer Hospital, Chinese Academy of Medical Sciences and Peking Union Medical College, Beijing, China.

**Keywords:** distant metastasis, gastric, lymphatic metastasis, neuroendocrine tumors, prognosis

## Abstract

Gastric neuroendocrine tumors (NETs) are a group of rare diseases, and we aimed to describe their clinicopathological features and explore the risk factors affecting metastatic disease. A retrospective analysis of 1480 patients with gastric NETs who underwent surgical resection from the Surveillance, Epidemiology, and End Results database between 2010 and 2019 was performed. Logistic regression analyses were conducted to identify the independent risk factors for regional lymph node metastasis and distant metastasis. The Kaplan‒Meier method was used to calculate cancer-specific survival rates. Cumulative incidence of cancer-specific mortality was calculated by a competing risk model. Of the 1480 patients, 1142 (77.2%) had G1 NETs, and 338 (22.8%) had G2 NETs. With regard to tumor size, 950 (64.2%) had primary neoplasms smaller than 1 cm, 302 (20.4%) had neoplasms within 1 to 2 cm in size, and 228 (15.4%) had neoplasms larger than or equal to 2 cm. Lymph node and distant metastases were identified through a comprehensive evaluation of imaging examinations. In total, 90 (6.1%) and 40 (2.7%) patients had regional lymph node metastasis and distant metastasis, respectively. Logistic regression analysis revealed that sex (*P* = .011), race (*P* = .049), tumor size (*P* < .001), and T stage (*P* < 001) were the independent risk factors for regional lymph node metastasis, while tumor grade (*P* = .033) and regional lymph node metastasis (*P* < .001) were the independent risk factors for distant metastasis. Survival analysis demonstrated that patients with gastric NETs had a favorable prognosis, with 3-year, 5-year, and 10-year cancer-specific survival rates of 97.4%, 96.1%, and 94.5%, respectively. Gastric NETs are generally slow-growing with favorable outcomes, though metastasis is possible. Tumor size and T stage primarily influence regional lymph node metastasis, whereas tumor grade and regional lymph node involvement are key risks for distant metastasis.

## 1. Introduction

Neuroendocrine neoplasms (NENs) are a rare and highly heterogeneous group of tumors originating from the diffuse neuroendocrine cell system. A subset of these neoplasms is characterized by the secretion of biologically active amines or peptide hormones.^[1]^ NENs can occur in multiple organs throughout the body, with the digestive system being the most common site, accounting for approximately 55% of all NEN cases.^[2]^ Gastric NENs represent 7% of all NENs, with an incidence rate of approximately 3 per 1 million. In recent years, the detection rate of this disease has significantly increased with the advancement of endoscopic techniques.^[3]^ According to the grading criteria for NEN in the 2022 World Health Organization Classification of NENs, gastric NENs can be classified as well-differentiated gastric neuroendocrine tumors (NETs) and poorly differentiated gastric neuroendocrine carcinomas (NECs).^[4]^

Gastric NETs are vastly more common than NECs, accounting for approximately 80% of all gastric NENs.^[5]^ Gastric NETs can be categorized into 3 distinct types based on cellular origin, pathogenesis, and associated underlying diseases. Type 1 gastric NETs are the most prevalent, comprising 80% to 90% of cases. In contrast, type 2 gastric NETs are the least prevalent, representing only 5% to 7% of cases, while type 3 gastric NETs constitute 10% to 15%. Both type 1 and type 2 gastric NETs originate from enterochromaffin-like cells and are strongly associated with elevated serum gastrin levels. Type 3 gastric NETs, however, arise from all neuroendocrine cells present within the stomach.^[1,6]^ Based on routine clinical experience, gastric NETs are usually characterized as diminutive, benign, early, and indolent lesions with extremely low risks of nodal and distant metastasis. Therefore, the use of radiology or nuclear medicine imaging is not recommended by guidelines, and endoscopic resection is often performed according to various expert consensuses, and favorable clinical outcomes have been confirmed by multiple clinical studies^[7,8]^ However, regional nodal and distant metastases are still occasionally reported in the literature, and the reported smallest primary lesion with nodal metastasis is only 0.3 × 0.3 cm in size.^[9-11]^ Therefore, the simple choice of endoscopic resection without cross-sectional diagnostic imaging evaluation of the status of regional lymph nodes and distant organs might lead to ignorance of metastatic disease. In this study, we aimed to delineate the rates of metastatic disease in gastric NETs and explore their risk factors for nodal and distant metastasis.

## 2. Methods and materials

### 2.1. Patients and data collection

We followed the methods of Wang and Liu.^[12]^ This study was approved by the Ethics Committee of the Xinxiang Central Hospital and adhered to the principles outlined in the Declaration of Helsinki by the World Medical Association. The Surveillance, Epidemiology, and End Results (SEER) database (www.seer.cancer.gov) was used to conduct a retrospective review of patients with gastric NETs and incidence data from the SEER database. Data from 17 registries, November 2021 Sub (2000–2019), were downloaded using SEER*Stat software (version 8.4.0.1; Surveillance Research Program, National Cancer Institute). The variable “AYA site recode 2020 Revision” was employed to identify cases of gastric NETs. The ICD-O-3 histology codes were not used in the case selection for this study. Participants included in this study were those whose classification under the “AYA site recode 2020 Revision” was “9.3.2.1.1 Neuroendocrine tumor (NET).” Tumor grading was determined based on the degree of differentiation, as specified in the SEER database using the following ICD-O-3 criteria: Grade I for well-differentiated tumors, Grade II for moderately differentiated tumors, Grade III for poorly differentiated tumors, and Grade IV for undifferentiated (anaplastic) tumors.^[4,13]^ The specific SEER variables employed to distinguish between G1 and G2 in this study comprised “Grade Recode (thru 2017),” “Grade Clinical (2018+),” and “Grade Pathological (2018+).” The SEER database comprehensively documents the transitions in TNM staging over various time periods. In this study, we extracted T-stage information for tumors from 2010 to 2015 using the variable “Derived AJCC T, 7th ed (2010–2015).” For data spanning 2016 to 2017, the extraction was based on the variable “Derived SEER Combined T (2016–2017).” Similarly, for the period from 2018 to 2019, we utilized the variable “Derived EOD 2018 T (2018+)” to obtain the relevant T-stage information. The N-stage information was derived from the variables “Derived AJCC N, 7th ed (2010–2015),” “Derived SEER Combined N (2016–2017),” and “Derived EOD 2018 N (2018+).” Likewise, the M-stage information was sourced from the variables “Derived AJCC M, 7th ed (2010–2015),” “Derived SEER Combined M (2016–2017),” and “Derived EOD 2018 M (2018+).” The evaluation of T, N and M stages was identified through a comprehensive evaluation of imaging examinations. Information regarding surgical methods was obtained through the variables “RX Summ-Surg Prim Site (1998+),” “RX Summ-Scope Reg LN Sur (2003+)” and “RX Summ-Surg Oth Reg/Dis (2003+).” All SEER variables used in this study were presented in the Table S1, Supplemental Digital Content 1.

The inclusion criteria were as follows: all patients who underwent surgical resection of their primary tumor, including both endoscopic resection and radical surgery, all patients who were pathologically diagnosed with G1 and G2 NENs, all patients whose NENs were located in the stomach, all patients whose NENs were confirmed to be NETs, and patients who were diagnosed after 2010. The exclusion criteria were as follows: NENs located in the rest of the gastrointestinal tract, NENs categorized as G3 or G4 grade, and incomplete data. As the SEER database did not distinguish G3 NETs from NECs, G3 NETs were not included in this study. According to our inclusion and exclusion criteria, 1480 eligible patients were ultimately included in our study (Fig. 1). The comparison of variables between the included patients and the excluded patients was presented in Table S2, Supplemental Digital Content 2.

**Figure 1.**
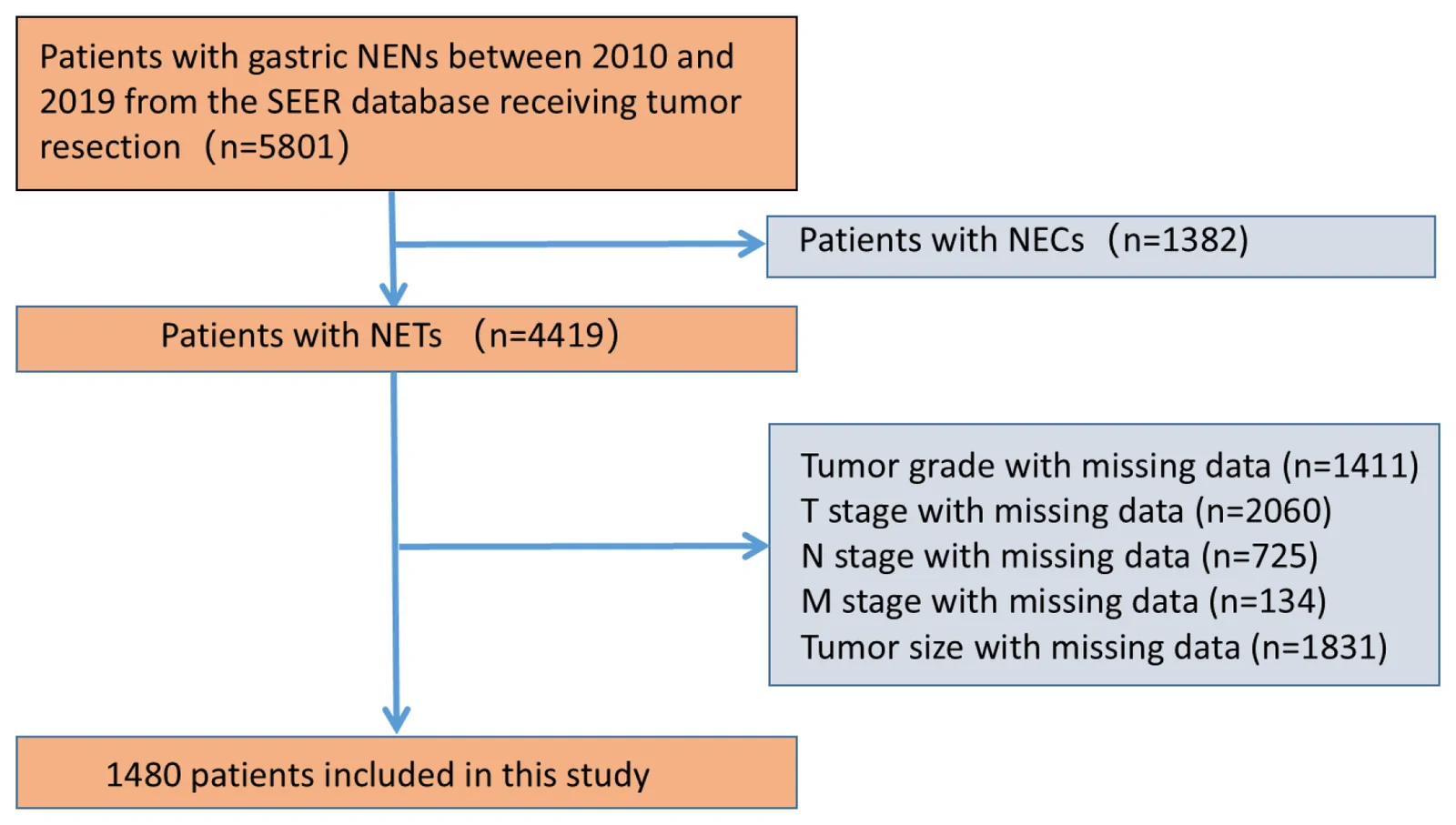
Flowchart of the study. NEC = neuroendocrine carcinoma, NEN = neuroendocrine neoplasm, NET = neuroendocrine tumor, SEER = Surveillance, Epidemiology, and End Results.

### 2.2. Statistical analysis

Quantitative data are presented as medians with interquartile ranges (IQRs). Qualitative and ordinal variables are presented as numbers with percentages. Univariate and multivariate logistic regression analysis was performed to screen for possible risk factors for regional lymph node metastasis. Multivariable logistic regression with a Firth correction was used to identify risk factors for distant metastasis. Odds ratios and 95% confidence intervals were also calculated.

Survival analysis was performed, and cancer-specific survival (CSS) rates were calculated by generating Kaplan‒Meier curves. CSS was defined as the time from diagnosis to death caused by gastric NETs. The survival duration is extracted from the SEER variable “Survival months,” whereas the survival status is determined using the SEER variables “Vital status recode (study cutoff used)” and “SEER cause-specific death classification.” The CSS rates of different groups were compared through the log-rank test. Furthermore, the cumulative incidence of cancer-specific mortality (CSM) was assessed using a competing risks model, with death from other causes considered a competing event to CSM. Gray’s test was employed to evaluate the differences in CSM between groups. All the statistical analyses were performed using the Statistical Package for the Social Sciences (SPSS version 24.0, IBM Corp., Armonk) and R, version 4.5.1 (R Project for Statistical Computing). Statistical significance was set at a 2-sided *P* value < .05.

## 3. Results

### 3.1. Patient characteristics

The basic clinical and pathological manifestations of the cohort are presented in Table 1. In total, 1480 eligible patients with a median age of 61 (IQR: 51, 69) years were included in this study. Of these patients, 533 (36.0%) were male, and 947 (64.0%) were female. Most (77.2%) patients were white, 217 (14.7%) were black, and 120 (8.1%) were from other ethnic groups. A total of 68 individuals (4.6%) presented with tumors located in the cardia, 139 individuals (9.4%) had tumors in the gastric fundus, 542 individuals (36.6%) had tumors in the gastric body, 109 individuals (7.4%) had tumors in the antrum, and 19 individuals (1.3%) had tumors in the pylorus. Additionally, 88 individuals (5.9%) had tumors situated in the lesser curvature, 100 individuals (6.8%) had tumors in the greater curvature, and 43 individuals (2.9%) had tumors in overlapping gastric lesions.

**Table 1:** The clinical and pathological features of patients.

Variable	Patient (n = 1480)
Gender	
Male	533 (36.0%)
Female	947 (64.0%)
Age (yr)	
Median (IQR)	61 (51, 69)
Race	
White	1143 (77.2%)
Black	217 (14.7%)
Other	120 (8.1%)
Tumor location	
Cardia	68 (4.6%)
Fundus of stomach	139 (9.4%)
Body of stomach	542 (36.6%)
Gastric antrum	109 (7.4%)
Pylorus	19 (1.3%)
Lesser curvature of stomach	88 (5.9%)
Greater curvature of stomach	100 (6.8%)
Overlapping lesion of stomach	43 (2.9%)
Other	372 (25.1%)
Surgical method	
Endoscopic resection	1261 (85.2%)
Gastrectomy	219 (14.8%)
Tumor grade	
G1	1142 (77.2%)
G2	338 (22.8%)
Tumor size (cm)	
<1 cm	950 (64.2%)
1–2 cm	302 (20.4%)
≥2 cm	228 (15.4%)
T staging	
T1	1013 (68.4%)
T2	388 (26.2%)
T3	54 (3.6%)
T4	25 (1.7%)
Regional lymph nodes metastasis	
Negative	1390 (93.9%)
Positive	90 (6.1%)
Distant metastasis	
Negative	1440 (97.3%)
Positive	40 (2.7%)
Population	
Metropolitan	1290 (87.2%)
Rural/urban	190 (12.8%)
Median income ($)	
<49,999	177 (12.0%)
50,000–74,999	543 (36.7%)
≥75,000	760 (51.4%)

IQR = interquartile range.

In the context of surgical method selection, a total of 1261 patients (85.2%) underwent endoscopic resection, while the remaining 219 patients (14.8%) underwent gastrectomy with accompanying regional lymph node dissection. Among those who underwent regional lymph node dissection, a median of 22 lymph nodes (IQR: 16, 28) were identified. Concerning tumor grade, 1142 patients (77.2%) were diagnosed with G1 gastric NETs, whereas 338 patients (22.8%) had G2 gastric NETs. In terms of primary tumor size, the majority of patients (64.2%) presented with tumors measuring <1 cm, 302 patients (20.4%) had tumors ranging from 1 to 2 cm, and 228 patients (15.4%) had tumors measuring 2 cm or larger. Regarding the T stage classification, most patients (68.4%) were categorized with T1 NETs, 388 patients (26.2%) with T2 NETs, 54 patients (3.6%) with T3 NETs, and 25 patients (1.7%) with T4 NETs. Finally, 90 patients (6.1%) exhibited regional lymph node metastasis, and 40 patients (2.7%) presented with distant metastasis.

### 3.2. Risk factors for regional lymph node metastasis

The independent risk factors for regional lymph node metastasis were identified through univariate and multivariate logistic regression analyses (Table 2). Univariate analysis revealed that sex (*P* < .001), race (*P* = .002), tumor grade (*P* = .001), tumor size (*P* < .001), and tumor T stage (*P* < .001) were significantly associated with the presence of regional lymph node metastasis. The multivariate analysis showed that sex (*P* = .011), race (*P* = .049), tumor size (*P* < .001), and tumor T stage (*P* < .001) were confirmed to be independent risk factors for regional lymph node metastasis.

**Table 2:** Univariate and multivariate logistic regression analysis of regional lymph nodes metastasis.

	Univariate analysis		Multivariate analysis	
	OR (95% CI)	*P*	OR (95% CI)	*P*
Gender		<.001		.011
Male	1		1	
Female	0.387 (0.251–0.596)		0.519 (0.314–0.858)	
Age (yr)		.094		.159
<60	1		1	
≥60	0.694 (0.452–1.064)		0.696 (0.421–1.152)	
Race		.002		.049
White	1			
Black	2.436 (1.487–3.990)		1.844 (1.023–3.321)	
Other	1.159 (0.517–2.599)		0.603 (0.229–1.591)	
Tumor location		.792		.614
Upper	1		1	
Middle	0.838 (0.436–1.606)		1.087 (0.500–2.363)	
Lower	1.168 (0.503–2.715)		0.600 (0.220–1.637)	
Other	0.849 (0.447–1.613)		0.899 (0.421–1.919)	
Tumor grade		.001		.449
G1	1		1	
G2	2.169 (1.390–3.384)		1.229 (0.721–2.094)	
Tumor size (cm)		<.001		<.001
<1 cm	1		1	
1–2 cm	6.379 (3.039–13.392)		3.387 (1.223–9.379)	
≥2 cm	29.124 (14.979–56.628)		9.999 (3.330–30.020)	
T staging		<.001		<.001
T1	1		1	
T2	7.974 (4.278–14.863)		1.587 (0.592–4.251)	
T3	49.058 (23.014-104.575)		9.100 (3.013–27.485)	
T4	107.036 (41.050–279.090)		13.791 (3.699–51.418)	

CI = confidence interval, OR = odds ratio.

Based on the results of multivariate analysis, tumor size and T stage demonstrated the most significant correlation with regional lymph node metastasis, and the probability of regional lymph node metastasis in patients based on tumor size and T stage was calculated and presented in Table 3. For patients with tumors <1 cm, 1 to 2 cm, and ≥ 2 cm, the risk of regional lymph node metastasis was 1.2%, 7.0%, and 25.4%, respectively. Patients with T1 and T2 NETs <1 cm had the lowest probability of regional lymph node metastasis (1.2%), and more than half of the patients (52.7%) with T3 and T4 NETs ≥ 2 cm presented with regional lymph node metastasis.

**Table 3:** The probabilities of regional lymph node metastasis stratified by tumor size and T stage.

	< 1 cm (n = 950)		1–2 cm (n = 302)		≥2 cm (n = 228)	
	Ratio	95% CI	Ratio	95% CI	Ratio	95% CI
T1 and T2	11/944 (1.2%)	0.5–1.9%	13/284 (4.6%)	2.1–7.0%	29/173 (16.8%)	11.1–22.4%
T3 and T4	0/6	0	8/18 (44.4%)	19.0–69.9%	29/55 (52.7%)	39.1–66.3%
In total	11/950 (1.2%)	0.5–1.8%	21/302 (7.0%)	4.1–9.8%	58/228 (25.4%)	19.7–31.1%

CI = confidence interval.

### 3.3. Risk factors for distant metastasis

The multivariate logistic analyses, incorporating a Firth correction for distant metastasis, were comprehensively presented in Table 4. The multivariate analysis revealed that tumor grade (*P* = .033) and regional lymph node metastasis (*P* < .001) serve as independent risk factors for distant metastasis. Furthermore, tumors situated in the lower region of the stomach demonstrated a higher risk of distant metastasis compared to those in upper regions (Adjusted odds ratio = 3.776, *P* = .039). The limited sample size for patients with distant metastasis, tumors exceeding 2 cm, and those classified as stage T4 introduced significant uncertainty in the estimation of the overall parameters, leading to excessively wide confidence intervals. Subsequently, the probabilities of distant metastasis for patients were computed and outlined in Table 5. Patients diagnosed with G1 NETs and without regional lymph node metastasis exhibited the lowest risk of distant metastasis at 1.0%, whereas those with G2 NETs and regional lymph node metastasis demonstrated the highest risk, at 32.4%.

**Table 4:** Multivariate logistic regression analysis with a Firth correction for distant metastasis.

	Adjusted OR	95% CI	P
Gender			
Male	1		
Female	1.566	0.708–3.670	0.273
Age (yr)			
<60	1		
≥60	0.776	0.364–1.648	0.507
Race			
White	1		
Black	1.051	0.404–2.500	0.914
Other	1.406	0.359–4.439	0.599
Tumor location			
Upper	1		
Middle	1.226	0.382–4.526	0.740
Lower	3.776	1.068–15.003	0.039
Unknown	1.166	0.375–4.212	0.798
Tumor grade			
G1	1		
G2	2.321	1.071–4.984	0.033
Tumor size (cm)			
<1 cm	1		
1–2 cm	0.260	0.014–2.476	0.295
≥2 cm	3.693	0.474–40.791	0.258
T staging			
T1	1		
T2	1.043	0.098–8.147	0.974
T3	0.997	0.073–10.360	0.999
T4	9.526	0.762–102.411	0.084
Regional lymph nodes metastasis			
Negative	1		
Positive	8.093	3.343–20.065	<0.001

CI = confidence interval, OR = odds ratio.

**Table 5:** The probabilities of distant metastasis stratified by tumor grade and status of regional lymph nodes.

	G1 (n = 1142)		G2 (n = 338)	
	Ratio	95% CI	Ratio	95% CI
N0	11/1086 (1.0%)	0.4–1.6%	7/304 (2.3%)	0.6–4%
N1	11/56 (19.6%)	8.9–30.4%	11/34 (32.4%)	15.8–48.9%
In total	22/1142 (1.9%)	1.1–2.7%	18/338 (5.3%)	2.8–7.7%

CI = confidence interval.

### 3.4. Survival analysis

In this study, patients diagnosed with gastric NETs demonstrated favorable oncological outcomes, with only 46 out of 1480 patients (3.1%) succumbing to tumor progression. The CSS rates for the entire cohort at 3, 5, and 10 years were 97.4%, 96.1%, and 94.5%, respectively (Fig. 2A). Subsequently, we conducted a further prognostic evaluation distinguishing between patients without metastatic disease and those with either regional lymph node involvement or distant metastasis (Fig. 2B). For patients without metastatic disease, the 3-year, 5-year, and 10-year CSS rates were 98.2%, 97.2%, and 95.5%, respectively. Conversely, for patients with regional lymph node involvement or distant metastasis, the corresponding CSS rates were 87.2%, 82.1%, and 82.1%. In contrast, patients with regional lymph node involvement or distant metastasis exhibited significantly lower CSS rates compared to those without metastatic disease (*P* < .001). The competing risk model further corroborated these findings, indicating that patients with regional lymph node involvement or distant metastasis had significantly higher CSM rates than those without metastatic disease (*P* < .001; Fig. 3).

**Figure 2.**
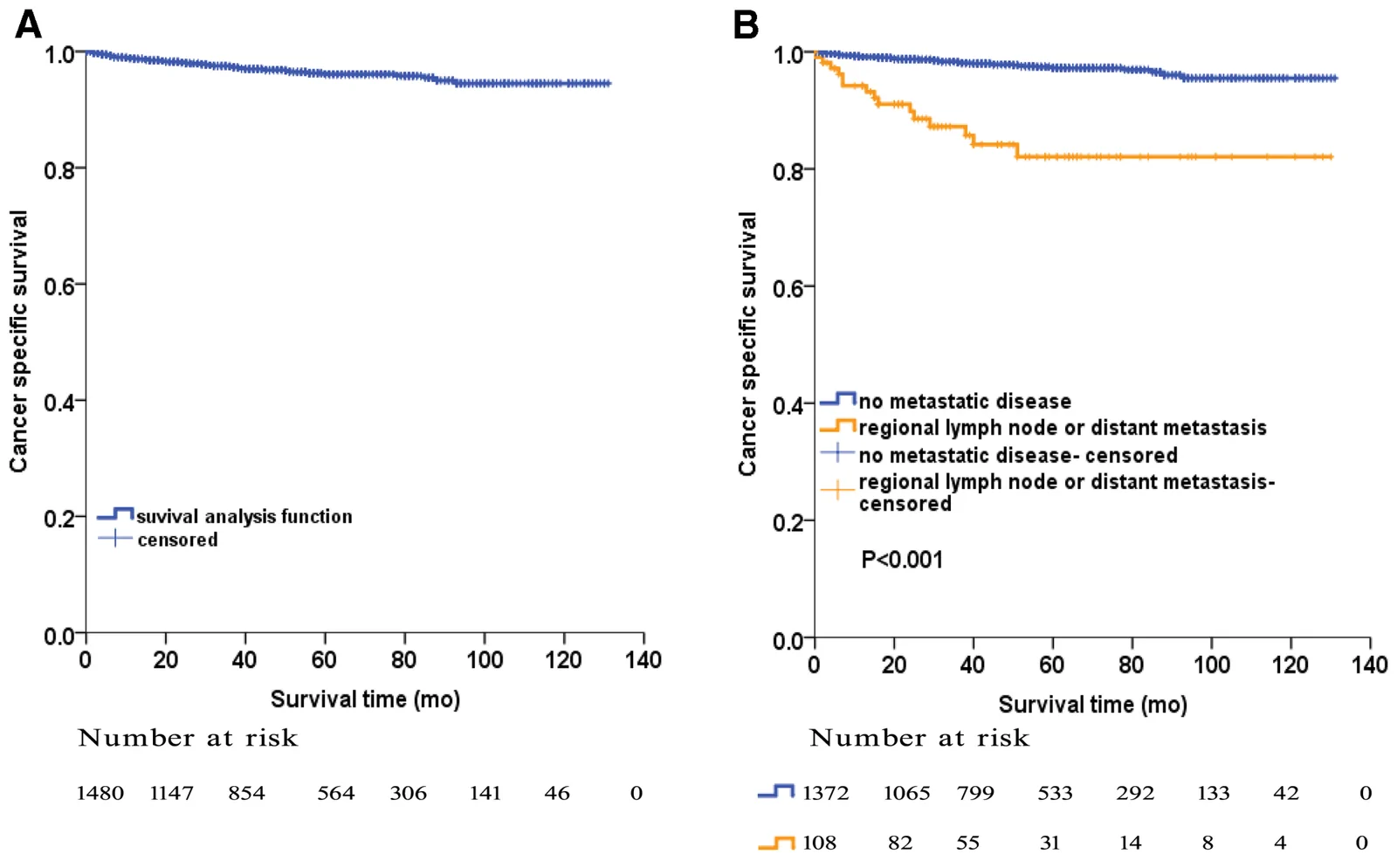
The cancer-specific survival (CSS) rates of well-differentiated gastric NETs. (A) The CSS rates of the whole cohort; (b) the CSS rates of patients without metastatic disease and with either regional lymph node metastasis or distant metastasis. CSS = cancer-specific survival, NET = neuroendocrine tumor.

**Figure 3.**
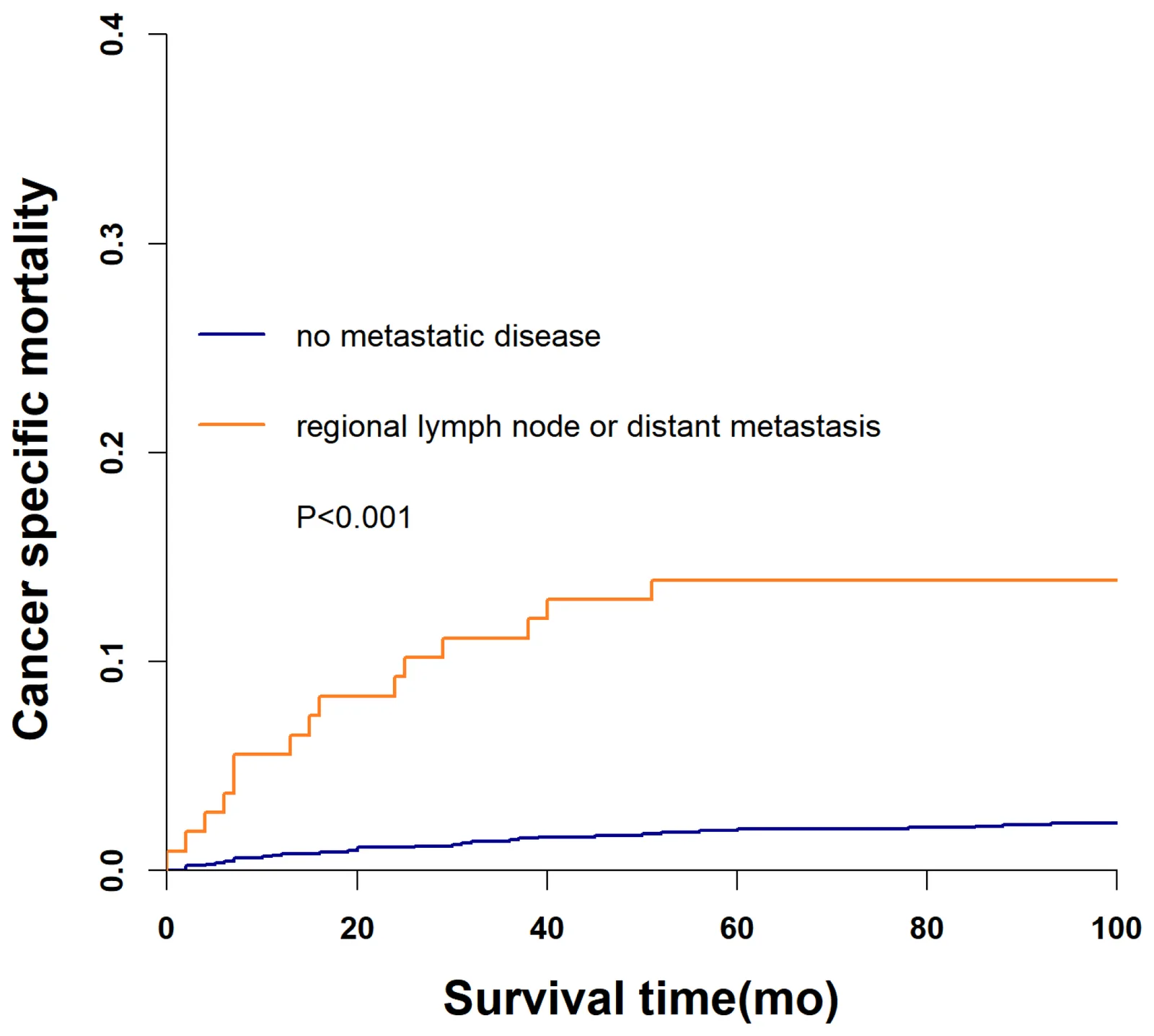
Cancer-specific mortality of patients from competing risk model.

## 4. Discussion

Our study suggests that, although common clinical experience indicates that gastric NETs may be a type of benign tumor, with endoscopic resection potentially achieving a curative effect, a small proportion of tumors may still have latent malignancy and exhibit regional lymph node metastasis or even distant metastasis, which should not be overlooked. This is particularly true for tumors that are larger, of higher grade, and have infiltrated beyond the submucosa. Endoscopic ultrasonography is crucial for the locoregional assessment of type 1 NETs; however, further investigation is required to establish the size threshold that justifies its use. Conventional imaging modalities, including computed tomography (CT) and magnetic resonance imaging (MRI), offer limited utility in terms of cost-effectiveness for detecting small type 1 and type 2 gastric tumors. Nonetheless, these techniques are essential for staging advanced neoplasms and type 3 NETs.^[14,15]^

Due to the low incidence of gastric NETs, there have been very few clinical studies reported in the past. Bethineedi et al reported a case of gastric G1 NET with multiple liver metastatic lesions.^[10]^ Jung et al reported 1 case with distant metastasis and 2 cases with regional lymph node metastasis of gastric NETs, attempting to explore the risk factors for tumor metastasis; however, no positive risk factors were found due to the small sample size.^[16]^ Li et al reported 82 cases of gastric NETs with regional lymph node metastasis, finding that albumin, carcinoembryonic antigen, tumor location, and Ki-67 expression level correlate with the risk of lymph node metastasis in patients with gastric NETs.^[17]^ Wang et al reported on 1153 cases of well-differentiated gastric NETs, with 63 (5.4%) patients developing metastatic lesions.^[18]^ This is consistent with our findings.

The clinicopathological manifestations of gastric NETs are significantly different from those of gastric NECs, although both are gastric NENs. Gastric NECs are highly aggressive and malignant with high probabilities of widespread metastasis and dismal clinical outcomes.^[19,20]^ Gastric NETs are a group of indolent diseases with favorable prognoses; they are often incidentally detected by gastric endoscopy and tend to be characterized as small (<10 mm) polypoid lesions or, more frequently, as smooth hemispherical submucosal lesions that may appear yellow or red in color.^[21]^ The respective molecular features of gastric NETs and NECs might help to explain their large differences. Gastric NETs often present mutations in the MEN1, DAXX, and ATRX genes, whereas gastric NECs often harbor mutations in the TP53 and RB1 genes, which are also usually detected in gastric adenocarcinomas.^[22-24]^

Given their well-differentiated pathological features and indolent clinical presentations, most Chinese physicians consider gastric NETs to be benign diseases that do not typically involve regional lymph node metastasis or distant metastasis, especially in areas with low population density and in hospitals with low patient volume. Endoscopic resection is usually performed by most physicians for these patients, even without adequate evaluation of the status of regional lymph nodes and distant organs. Moreover, regular postoperative reexamination often fails because of the ignorance of the underlying possibility of recurrence or metastasis. In our study, we utilized a large public database and revealed that 6.1% and 2.7% of gastric NETs exhibited regional lymph node metastasis and distant metastasis, respectively. This finding suggests the malignant potential of gastric NETs. To avoid missing metastatic lesions and potential recurrence after surgery, comprehensive preoperative staging evaluation and regular postoperative follow-up are essential for patients with risk factors.^[25]^ Endoscopic ultrasonography is useful for assessing the depth of the tumor and its location within the layers of the gastric wall.^[26]^ CT or MRI can help to provide additional information on local spread and distant metastasis.^[27]^

Tumor size is a determining factor in management strategies, as the risk of metastasis significantly increases with tumor size. However, the optimal threshold of tumor size for decision-making has always been controversial.^[26]^ For gastric NETs < 1 cm, noninterventional endoscopic surveillance programs or endoscopic resection have both been widely recommended.^[1,28]^ Our study indicated that these patients had probabilities of regional lymph node metastasis and distant metastasis of 1.2% and 0.9%, respectively. Considering the potential malignancy of these tumors, despite the low risk of metastatic disease, we still recommend tumor resection to prevent tumor progression and the occurrence of metastatic lesions. However, there is significant disagreement in treatment recommendations for gastric NETs with diameters between 1 and 2 cm. The National Comprehensive Cancer Network guidelines still recommend simple surveillance or endoscopic resection, while the guidelines from the Italian Society of Digestive Endoscopy suggest endoscopic resection to mitigate the potential risk of further growth and subsequent metastasis.^[7,21]^ Our study indicated 7.0% and 0.3% probabilities of regional lymph node metastasis and distant metastasis, respectively, for gastric NETs 1 to 2 cm in size. Owing to the relatively high risk of local spread, we suggest that endoscopic resection might be a reasonable choice. Moreover, careful and thorough preoperative examinations must be conducted to avoid missing metastatic lymph node lesions. For patients with gastric NETs ≥ 2 cm, there is controversy among different guidelines on whether to choose endoscopic resection or radical surgical resection. The National Comprehensive Cancer Network and European Neuroendocrine Tumor Society Guidelines recommend endoscopic resection after excluding invasion extending beyond the submucosa, the presence of lymph node metastasis, and distant metastasis.^[14,21]^ However, the Italian guidelines discourage endoscopic resection and recommend radical surgical resection with lymph node dissection.^[7]^ In our study, we found that patients with gastric NETs ≥ 2 cm had a 25.4% and 13.2% risk of regional lymph node metastasis and distant metastasis, respectively, which cannot be ignored. Owing to the high risk of potential existing metastatic lesions, we believe that radical surgical resection might be a more reasonable choice. Considering the high risk of distant metastasis, CT or MRI must be performed to evaluate the status of distant organs, especially the liver and distant lymph nodes. When necessary, if conventional methods are unable to identify whether distant organs have metastatic lesions, consideration should be given to performing somatostatin receptor scintigraphy or 68Ga-labeled positron emission tomography tracers (68Ga DOTATOC, DOTANOC, and DOTATATE) at a qualified medical center to clarify the nature of suspicious distant lesions.^[14,29,30]^ If distant metastasis is confirmed by radiological examinations, there is currently no high-level evidence in evidence-based medicine to guide the choice between surgical treatment or systemic therapy, and a multidisciplinary discussion is needed to formulate a treatment plan.

Our study is subject to several limitations. First, it is a retrospective observational study, which may introduce information bias. In our effort to include patients with pathologically confirmed NETs, we restricted our sample to those who had undergone surgical intervention. Additionally, a significant number of patients were excluded due to incomplete data, potentially leading to selection bias due to differences in characteristics between excluded and included patients. Second, the management of gastric NETs is highly dependent on clinical subtype and the presence of hypergastrinemia. However, the SEER database does not provide information on hypergastrinemia, atrophic gastritis, the Ki67 index, or the mitotic index, limiting our ability to differentiate between these subtypes. Thirdly, only 219 patients (14.8%) underwent gastrectomy with accompanying regional lymph node dissection, which might lead to understaging as the remaining 1261 patients (85.2%) underwent only endoscopic resection. Lastly, the SEER database does not distinguish between NET G3 and NEC, which led us to include only NET G1 and NET G2 in our study, thereby excluding NET G3.

In conclusion, gastric NETs represent a category of indolent diseases generally associated with favorable oncological outcomes. However, their potential for aggressiveness should not be underestimated by clinicians. Analysis of data from patients who underwent surgical resection, as recorded in the SEER database, indicates that tumor size and T stage are the primary risk factors for regional lymph node metastasis. Additionally, tumor grade and regional lymph node metastasis are significantly correlated with distant metastasis. It is imperative for physicians to consider these factors comprehensively when assessing the risk of metastatic lesions prior to developing a treatment plan.

## Author contributions

**Conceptualization:** Aimin Yue.

**Data curation:** Yue Zhang.

**Formal analysis:** Lu Liu.

**Methodology:** Lu Liu.

**Software:** Lu Liu.

**Writing – original draft:** Yuhou Shen.

**Writing – review & editing:** Aimin Yue.




