# Supplementary material for: Risk factor analysis of nodal and distant metastasis for G1 and G2 gastric neuroendocrine tumors
Source: Medicine (Baltimore). 2026 Oct 30;105(44):e51423. doi: 10.1097/MD.0000000000051423 (PMC13727220; doi:10.1097/MD.0000000000051423)
Supplement: Supplementary file 1 [file medi-105-e51423-s001.docx]

Table S1. All SEER variables used in this study.

| SEER variable names | Coding definitions |
| --- | --- |
| Patient ID | This field is used to uniquely iActual age in years dentify a person. |
| Age recode with single ages and 90+ | 000-084:Actual age in years;  85:85+ years  127:unknown age |
| Sex | 1:Male; 2: Female; 3:Not stated (unknown) |
| Year of diagnosis | The year of diagnosis is the year the tumor was first diagnosed by a recognized medical practitioner, whether clinically or microscopically confirmed. Allowable values YYYY = 1975 – forward |
| Race recode (W, B, AI, API) | 1:White  2:Black  3:American Indian/Alaska Native  4:Asian or Pacific Islander  7:Other unspecified (1991+)  9:Unknown |
| Primary Site - labeled | This provides the primary site code in ICD-O-3 and a descriptive primary site label. Note that the label is the preferred ICD-O-3 bolded name and there may be other sites or sub-sites included in the code but not reflected in the preferred term. Refer to ICD-O-3 for further information. Cases with years of diagnosis before 1992 were converted to ICD-O-3 from earlier versions. |
| Grade Recode (thru 2017) | 1:Grade I; grade i; grade 1; well differentiated; differentiated, NOS  2:Grade II; grade ii; grade 2; moderately differentiated; moderately differentiated; intermediate differentiation  3:Grade III; grade iii; grade 3; poorly differentiated; differentiated  4:Grade IV; grade iv; grade 4; undifferentiated; anaplastic  5:T-cell; T-precursor  6:B-cell; Pre-B; B-Precursor  7:Null cell; Non T-non B;  8:N K cell (natural killer cell)  9:cell type not determined, not stated or not applicable |
| Grade Pathological (2018+) | Refer to the most recent Grade Manual for additional site-specific instructions. https://apps.naaccr.org/ssdi/list/ |
| ICD-O-3 Hist/behav, malignant | Labeled version of ICD-O-3 values for malignant tumors. All non-malignant tumors are grouped into one value. See SEER*Stat dictionary for labels. |
| Derived AJCC Stage Group, 7th ed (2010-2015) | 000: Stage 0  010: Stage 0a  020: Stage 0is  100: Stage I  110: Stage I NOS  120: Stage IA  130: Stage IA1  140: Stage IA2  121: Stage IA NOS  150: Stage IB  160: Stage IB1  170: Stage IB2  151: Stage IB NOS  180: Stage IC  190: Stage IS  230: Stage ISA (lymphoma only)  240: Stage ISB (lymphoma only)  200: Stage IEA (lymphoma only)  210: Stage IEB (lymphoma only)  220: Stage IE (lymphoma only)  300: Stage II  310: Stage II NOS  320: Stage IIA  321: Stage IIA NOS  322: Stage IIA1  323: Stage IIA NOS  330: Stage IIB  340: Stage IIC  350: Stage IIEA (lymphoma only)  360: Stage IIEB (lymphoma only)  370: Stage IIE (lymphoma only)  380: Stage IISA (lymphoma only)  390: Stage IISB (lymphoma only)  400: Stage IIS (lymphoma only)  410: Stage IIESA (lymphoma only)  420: Stage IIESB (lymphoma only)  430: Stage IIES (lymphoma only)  500: Stage III  510: Stage III NOS  520: Stage IIIA  530: Stage IIIB  540: Stage IIIC  541: Stage IIIC1  542: Stage IIIC2  550: Stage IIIEA (lymphoma only)  560: Stage IIIEB (lymphoma only)  570: Stage IIIE (lymphoma only)  580: Stage IIISA (lymphoma only)  590: Stage IIISB (lymphoma only)  600: Stage IIIS (lymphoma only)  610: Stage IIIESA (lymphoma only)  620: Stage IIIESB (lymphoma only)  630: Stage IIIES (lymphoma only)  700: Stage IV  710: Stage IV NOS  720: Stage IVA  730: Stage IVB  740: Stage IVC  888: Not applicable  900: Stage Occult  999: Stage Unknown  1022: Blank |
| Derived AJCC T, 7th ed (2010-2015) | 999: TX  000: T0  010: Ta  050: Tis  060: Tispu (Urethra only)  070: Tispd (Urethra only)  100: T1  110: T1mic  199: T1 NOS  120: T1a  130: T1a1  140: T1a2  150: T1b  160: T1b1  170: T1b2  180: T1c  181: T1d  200: T2  299: T2 NOS  210: T2a  211: T2a1  212: T2a2  213: T2a NOS  220: T2b  230: T2c  240: T2d  300: T3  399: T3 NOS  310: T3a  320: T3b  330: T3c  400: T4  499: T4 NOS  410: T4a  420: T4b  430: T4c  440: T4d  450: T4e  800: T1a NOS  810: T1b NOS  888: Not applicable  1022: Blank |
| Derived AJCC N, 7th ed (2010-2015) | 999: NX  000: N0  010: N0(i-)  020: N0(i+)  030: N0(mol-)  040: N0(mol+)  100: N1  199: N1 NOS  110: N1a  120: N1b  130: N1c  180: N1mi  200: N2  299: N2 NOS  210: N2a  220: N2b  230: N2c  300: N3  399: N3 NOS  310: N3a  320: N3b  330: N3c  400: N4  888: Not applicable  1022: Blank |
| Derived AJCC M, 7th ed (2010-2015) | 999: MX  000: M0  010: M0(i+)  100: M1  110: M1a  120: M1b  130: M1c  140: M1d  150: M1e  199: M1 NOS  888: Not applicable  1022: Blank |
| Derived EOD 2018 T (2018+) | See the most current version of EOD (https://staging.seer.cancer.gov/) for rules and site-specific codes and coding structures. |
| Derived EOD 2018 N (2018+) | See the most current version of EOD (https://staging.seer.cancer.gov/) for rules and site-specific codes and coding structures. |
| DERIVED EOD 2018 M (2018+) | See the most current version of EOD (https://staging.seer.cancer.gov/) for rules and site-specific codes and coding structures. |
| Derived EOD 2018 Stage Group (2018+) | See the most current version of EOD (https://staging.seer.cancer.gov/) for rules and site-specific codes and coding structures. |
| Derived SEER Cmb Stg Grp (2016-2017) | The results of the derived algorithmic calculation of Derived SEER Combined Stage Group. |
| Derived SEER Combined T (2016-2017) | The results of the derived algorithmic calculation of Derived SEER Combined T. |
| Derived SEER Combined N (2016-2017) | The results of the derived algorithmic calculation of Derived SEER Combined N. |
| Derived SEER Combined M (2016-2017) | The results of the derived algorithmic calculation of Derived SEER Combined M. |
| Derived SEER Combined T Src (2016-2017) | 1: Clinical  2: Pathologic  3: Clinical and pathologic information used  9: Unknown  14: Blank |
| Derived SEER Combined N Src (2016-2017) | 1: Clinical  2: Pathologic  3: Clinical and pathologic information used  9: Unknown  14: Blank |
| Derived SEER Combined M Src (2016-2017) | 1: Clinical  2: Pathologic  3: Clinical and pathologic information used  9: Unknown 14 Blank |
| RX Summ--Surg Prim Site (1998+) | 00:None; no surgical procedure of primary site; diagnosed at autopsy only  10-19: Site-specific codes. Tumor destruction; no pathologic specimen or unknown whether there is a pathologic specimen  20-80: Site-specific codes. Resection; pathologic specimen  90: Surgery, NOS. A surgical procedure to the primary site was done, but no information on the type of surgical procedure is provided.  98: Special codes for hematopoietic, reticuloendothelial, immunoproliferative, myeloproliferative diseases; ill defined sites; and unknown primaries (See site-specific codes for the sites and histologies), except death certificate only Blank  99: Unknown if surgery performed; death certificate only  127:Blank |
| RX Summ--Scope Reg LN Sur (2003+) | 0:No regional lymph nodes removed or aspirated; diagnosed at autopsy.  1: Biopsy or aspiration of regional lymph node, NOS  2: Sentinel lymph node biopsy [only]  3: Number of regional lymph nodes removed unknown, not stated; regional lymph nodes removed, NOS  4: 1 to 3 regional lymph nodes removed  5: 4 or more regional lymph nodes removed  6: Sentinel node biopsy and code 3, 4, or 5 at same time or timing not noted  7: Sentinel node biopsy and code 3, 4, or 5 at different times  9: Unknown or not applicable; death certificate only  14: Blank |
| RX Summ--Surg Oth Reg/Dis (2003+) | 0: None; diagnosed at autopsy  1: Nonprimary surgical procedure performed  2: Nonprimary surgical procedure to other regional sites  3: Non-primary surgical procedure to distant lymph node(s)  4: Nonprimary surgical procedure to distant site  5: Combination of codes 2, 3, or 4  9: Unknown; death certificate only  14: Blank |
| Reason no cancer-directed surgery | 0: Surgery performed  1*: Surgery not recommended  2*: Contraindicated due to other conditions; Autopsy Only case (1973-2002)  5: Patient died before recommended surgery  6: Unknown reason for no surgery  7*: Patient or patient’s guardian refused  8: Recommended, unknown if done  9: Unknown if surgery performed; Death Certificate Only case; Autopsy only case (2003+)  14: Blank |
| CS Tumor Size/Ext Eval (2004-2015) | Available for 2004-2015, but not required for the entire timeframe. Will be blank in cases not collected. For more information, see http://seer.cancer.gov/seerstat/variables/seer/ajcc-stage. |
| CS tumor size (2004-2015) | 000:Indicates no mass or no tumor found; for example, when a tumor of a stated primary site is not found, but the tumor has metastasized. 001-988: Exact size in millimeters  989: 989 millimeters or larger  990: Microscopic focus or foci only; no size of focus is given  991: Described as less than 1 cm  992: Described as less than 2 cm  993: Described as less than 3 cm  994: Described as less than 4 cm  995: Described as less than 5 cm  996-998: Site-specific codes where needed  999: Unknown; size not stated; not stated in patient record  888: Not applicable  1022: Blank |
| Tumor Size Summary (2016+) | 000: No mass/tumor found  001: 1 mm or described as less than 1 mm (0.1 cm or less than 0.1 cm)  002-988: Exact size in millimeters (2mm-988mm) (0.2 cm to 98.8 cm)  989: 989 millimeters or larger (98.9 cm or larger)  990: Microscopic focus or foci only and no size of focus is given  998: Alternate descriptions of tumor size for specific sites  999: Unknown; size not stated; Not documented in patient record; Size of tumor cannot be assessed; No excisional biopsy or tumor resection done; The only measurement(s) describes pieces or chips; Not applicable  1022: Blank |
| Regional nodes examined (1988+) | 00:No nodes were examined  01-89:Exact number of nodes examined  90:90 or more nodes were examined  95:No regional nodes were removed, but aspiration of regional nodes was performed  96:Regional lymph node removal was documented as a sampling, and the number of nodes is unknown/not stated  97:Regional lymph node removal was documented as a dissection, and the number of nodes is unknown/not stated  98:Regional lymph nodes were surgically removed, but the number of lymph nodes is unknown/not stated and not documented as a sampling or dissection; nodes were examined, but the number is unknown  99:Unknown whether nodes were examined; not applicable or negative; not stated in patient record  126:Blank |
| Regional nodes positive (1988+) | 00:All nodes examined are negative  01-89:Exact number of nodes positive  90:90 or more nodes are positive  95:Positive aspiration of lymph node(s) was performed  97:Positive nodes are documented, but number is unspecified  98:No nodes were examined  99:Unknown whether nodes are positive; not applicable; not stated in patient record  126:Blank |
| SEER cause-specific death classification | 0: Alive or dead of other cause  1: Dead  8: Dead (missing/unknown COD)  9: N/A |
| SEER other cause of death classification | 0: Alive or dead due to cancer  1: Dead  8: Dead (missing/unknown COD)  9: N/A |
| Survival months | 000-9998: 000-9998 months  9999: Unknown |
| Survival months flag | 0: Complete dates are available and there are 0 days of survival  1: Complete dates are available and there are more than 0 days of survival  2: Incomplete dates are available and there could be zero days of follow-up  3: Incomplete dates are available and there cannot be zero days of follow-up  8: Not calculated because a Death Certificate Only or Autopsy Only case  9: Unknown |
| Vital status recode (study cutoff used) | 1: Alive  0: Dead |
