# Supplementary material for: Risk factor analysis of nodal and distant metastasis for G1 and G2 gastric neuroendocrine tumors
Source: Medicine (Baltimore). 2026 Oct 30;105(44):e51423. doi: 10.1097/MD.0000000000051423 (PMC13727220; doi:10.1097/MD.0000000000051423)
Supplement: Supplementary file 2 [file medi-105-e51423-s002.docx]

Table S2. Comparison of variables between the included patients and the excluded patients.

|  | Included patients  (n=1480) | Excluded patients  (n=4321) | P |
| --- | --- | --- | --- |
| Gender |  |  | 0.279 |
| Male | 533 (36.0%) | 1489 (34.5%) |  |
| Female | 947 (64.0%) | 2832 (65.5%) |  |
| Age (yr) |  |  | 0.677 |
| < 60 | 660 (44.6%) | 1900 (44.0%) |  |
| ≥ 60 | 820 (55.4%) | 2421 (56.0%) |  |
| Race |  |  | 0.448 |
| White | 1143（77.2%） | 3305 (76.5%) |  |
| Black | 217（14.7%） | 619 (14.3%) |  |
| Other | 120（8.1%） | 397 (9.2%) |  |
| Tumor location |  |  | 0.105 |
| Cardia | 68 (4.6%) | 203 (4.7%) |  |
| Fundus of stomach | 139 (9.4%) | 402 (9.3%) |  |
| Body of stomach | 542 (36.6%) | 1623 (37.6%) |  |
| Gastric antrum | 109 (7.4%) | 335 (7.8%) |  |
| Pylorus | 19 (1.3%) | 67 (1.6%) |  |
| Lesser curvature of stomach | 88 (5.9%) | 198 (4.6%) |  |
| Greater curvature of stomach | 100 (6.8%) | 212 (4.9%) |  |
| Overlapping lesion of stomach | 43 (2.9%) | 136 (3.1%) |  |
| Unknown | 372 (25.1%) | 1145 (26.5%) |  |
